# Bis[1-benzyl-3-(quinolin-8-ylmeth­yl)-2,3-dihydro-1*H*-imidazol-2-yl]dibromido­palladium(II) acetonitrile disolvate

**DOI:** 10.1107/S1600536811000973

**Published:** 2011-01-15

**Authors:** Jiafeng Sun, Guixiang Wang

**Affiliations:** a Department of Materials and Chemical Engineering, Taishan University, Taian 271021, People’s Republic of China

## Abstract

In the title compound, [PdBr_2_(C_20_H_17_N_3_)_2_]·2CH_3_CN, the Pd atom, which lies on an inversion center, is four-coordinated in a square-planar geometry. The two imidazole rings are coplanar and nearly perpendicular to the plane formed by Pd, the coordinated imidazole C atom and one of the Br atoms, making a dihedral angle of 75.1 (2)°.

## Related literature

For *N*-heterocyclic carbenes, see: Herrmann (2002 ▶); Boeda & Nolan (2008 ▶). For related stuctures, see: Hahn *et al.* (2004 ▶); Huynh & Wu (2009 ▶). For the synthesis of the carbene ligand, see: Sun *et al.* (2009 ▶).
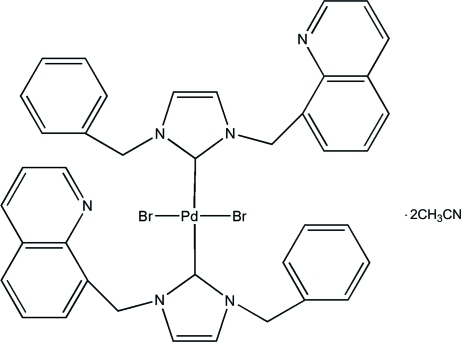


## Experimental

### 

#### Crystal data

[PdBr_2_(C_20_H_17_N_3_)_2_]·2C_2_H_3_N
*M* *_r_* = 947.06
Triclinic, 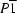

*a* = 8.179 (3) Å
*b* = 10.769 (4) Å
*c* = 11.928 (4) Å
α = 101.506 (5)°
β = 90.842 (5)°
γ = 107.944 (4)°
*V* = 976.1 (6) Å^3^
*Z* = 1
Mo *K*α radiation
μ = 2.57 mm^−1^
*T* = 173 K
0.30 × 0.24 × 0.12 mm

#### Data collection

Bruker SMART APEX CCD area-detector diffractometer
Absorption correction: multi-scan (*SADABS*; Bruker, 2002 ▶) *T*_min_ = 0.48, *T*_max_ = 0.74
5311 measured reflections
3728 independent reflections
2548 reflections with *I* > 2σ(*I*)
*R*_int_ = 0.100

#### Refinement

*R*[*F* ^2^ > 2σ(*F* ^2^)] = 0.060
*wR*(*F* ^2^) = 0.172
*S* = 1.01
3728 reflections
251 parameters
H-atom parameters constrained
Δρ_max_ = 1.65 e Å^−3^
Δρ_min_ = −1.90 e Å^−3^

### 

Data collection: *SMART* (Bruker, 2002 ▶); cell refinement: *SAINT* (Bruker, 2002 ▶); data reduction: *SAINT*; program(s) used to solve structure: *SHELXS97* (Sheldrick, 2008 ▶); program(s) used to refine structure: *SHELXL97* (Sheldrick, 2008 ▶); molecular graphics: *SHELXTL* (Sheldrick, 2008 ▶); software used to prepare material for publication: *SHELXTL*.

## Supplementary Material

Crystal structure: contains datablocks I, global. DOI: 10.1107/S1600536811000973/zq2083sup1.cif

Structure factors: contains datablocks I. DOI: 10.1107/S1600536811000973/zq2083Isup2.hkl

Additional supplementary materials:  crystallographic information; 3D view; checkCIF report

## Figures and Tables

**Fig. 1.**
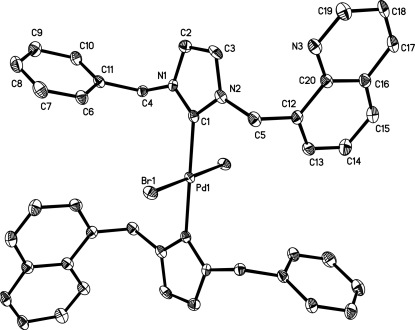
The molecular structure of title compound with atom labels and 30% probability displacement ellipsoids for non-H atoms (inversion symmetry-related atoms are not labeled and the solvent molecules are not included).

## supplementary crystallographic information

### Comment

In recent years, N-heterocyclic carbenes (NHCs) metal complexes have attracted much attention in coordination chemistry and homogeneous catalysis because NHCs are variable, can be easily modified and coordinated to various metals (Herrmann, 2002; Boeda *et al.*, 2008). Here we report the crystal structure of the title compound, C_40_H_34_Br_2_N_6_Pd.2CH_3_CN, which exhibits two N-heterocyclic carbenes and two bromo ligands coordinated to a Pd^II^ center.

The structure of the title compound is shown in Fig. 1. The Pd center is four-coordinated and displays a square planar coordination geometry. The Pd atom lies on an inversion center. The two imidazole rings are coplanar and nearly perpendicular to the plane formed by Pd, C1 and Br1, showing a dihedral angle of 75.1 (2)°. The Pd—C distance of 2.026 (7) Å agrees with distances found for similar biscarbene Pd complexes (Hahn *et al.*, 2004; Huynh & Wu, 2009). The asymmetric unit contains one solvent molecule of acetonitrile for one-half molecule of the metal-organic species.

### Experimental

1-Benzyl-3-(8-quinolylmethyl)imidazolium bromide was synthesized according to a literature method (Sun *et al.*, 2009). The resulting white solid (0.168 g, 0.44 mmol) was dissolved in acetonitrile (10 ml) and then palladium acetate (0.049 g, 0.22 mmol) was added. The mixture was stirred at refluxing temperature for 12 h and the solvent was removed under reduced pressure. The residue was then dissolved in distilled water and extracted with CH_2_Cl_2_. X-ray quality crystals of the title complex were obtained by slow diffusion of Et_2_O into its CH_3_CN solution.

### Refinement

The H atoms were included in the riding-model approximation, with C—H = 0.93 Å and *U*_iso_(H) = 1.2*U*_eq_(C) for the aromatic H atoms, and with C—H = 0.96 Å and *U*_iso_(H) = 1.5*U*_eq_(C) for the methyl H atoms.

### Crystal data

**Table:**

[PdBr_2_(C_20_H_17_N_3_)_2_]·2C_2_H_3_N	*Z* = 1
*M**_r_* = 947.06	*F*(000) = 476
Triclinic, *P*1	*D*_x_ = 1.611 Mg m^−^^3^
Hall symbol: -P 1	Mo *K*α radiation, λ = 0.71073 Å
*a* = 8.179 (3) Å	Cell parameters from 1719 reflections
*b* = 10.769 (4) Å	θ = 2.4–25.8°
*c* = 11.928 (4) Å	µ = 2.57 mm^−^^1^
α = 101.506 (5)°	*T* = 173 K
β = 90.842 (5)°	Block, pale yellow
γ = 107.944 (4)°	0.30 × 0.24 × 0.12 mm
*V* = 976.1 (6) Å^3^	

### Data collection

**Table:**

Bruker SMART APEX CCD area-detector diffractometer	3728 independent reflections
Radiation source: fine-focus sealed tube	2548 reflections with *I* > 2σ(*I*)
graphite	*R*_int_ = 0.100
φ and ω scans	θ_max_ = 26.0°, θ_min_ = 2.0°
Absorption correction: multi-scan (*SADABS*; Bruker, 2002)	*h* = −9→10
*T*_min_ = 0.48, *T*_max_ = 0.74	*k* = −13→12
5311 measured reflections	*l* = −7→14

### Refinement

**Table:**

Refinement on *F*^2^	Primary atom site location: structure-invariant direct methods
Least-squares matrix: full	Secondary atom site location: difference Fourier map
*R*[*F*^2^ > 2σ(*F*^2^)] = 0.060	Hydrogen site location: inferred from neighbouring sites
*wR*(*F*^2^) = 0.172	H-atom parameters constrained
*S* = 1.01	*w* = 1/[σ^2^(*F*_o_^2^) + (0.0905*P*)^2^] where *P* = (*F*_o_^2^ + 2*F*_c_^2^)/3
3728 reflections	(Δ/σ)_max_ < 0.001
251 parameters	Δρ_max_ = 1.65 e Å^−^^3^
0 restraints	Δρ_min_ = −1.90 e Å^−^^3^

### Special details

**Table:**

Geometry. All e.s.d.'s (except the e.s.d. in the dihedral angle between two l.s. planes) are estimated using the full covariance matrix. The cell e.s.d.'s are taken into account individually in the estimation of e.s.d.'s in distances, angles and torsion angles; correlations between e.s.d.'s in cell parameters are only used when they are defined by crystal symmetry. An approximate (isotropic) treatment of cell e.s.d.'s is used for estimating e.s.d.'s involving l.s. planes.
Refinement. Refinement of *F*^2^ against ALL reflections. The weighted *R*-factor *wR* and goodness of fit *S* are based on *F*^2^, conventional *R*-factors *R* are based on *F*, with *F* set to zero for negative *F*^2^. The threshold expression of *F*^2^ > σ(*F*^2^) is used only for calculating *R*-factors(gt) *etc*. and is not relevant to the choice of reflections for refinement. *R*-factors based on *F*^2^ are statistically about twice as large as those based on *F*, and *R*- factors based on ALL data will be even larger.

### Fractional atomic coordinates and isotropic or equivalent isotropic displacement parameters (Å^2^)

**Table:**

	*x*	*y*	*z*	*U*_iso_*/*U*_eq_	
Br1	0.58787 (9)	0.80115 (7)	−0.03850 (6)	0.0291 (2)	
C1	0.2797 (9)	0.8983 (6)	0.0605 (6)	0.0220 (14)	
C2	0.0012 (10)	0.7929 (7)	0.0814 (6)	0.0289 (16)	
H2	−0.1173	0.7541	0.0647	0.035*	
C3	0.0908 (10)	0.7998 (7)	0.1769 (6)	0.0313 (17)	
H3	0.0466	0.7677	0.2407	0.038*	
C4	0.0708 (9)	0.8680 (6)	−0.1036 (6)	0.0237 (15)	
H4A	0.1684	0.9302	−0.1295	0.028*	
H4B	−0.0233	0.9053	−0.0994	0.028*	
C5	0.4057 (9)	0.8970 (7)	0.2533 (6)	0.0298 (17)	
H5A	0.5140	0.9126	0.2176	0.036*	
H5B	0.3911	0.8219	0.2898	0.036*	
C6	0.1145 (10)	0.6504 (7)	−0.2030 (6)	0.0312 (17)	
H6	0.2149	0.6747	−0.1549	0.037*	
C7	0.0706 (11)	0.5328 (8)	−0.2815 (7)	0.041 (2)	
H7	0.1392	0.4775	−0.2868	0.049*	
C8	−0.0812 (11)	0.4964 (8)	−0.3550 (7)	0.040 (2)	
H8	−0.1128	0.4167	−0.4097	0.048*	
C9	−0.1824 (11)	0.5782 (8)	−0.3461 (6)	0.0371 (19)	
H9	−0.2830	0.5538	−0.3939	0.045*	
C10	−0.1315 (10)	0.7001 (8)	−0.2631 (6)	0.0307 (17)	
H10	−0.1984	0.7567	−0.2570	0.037*	
C11	0.0164 (9)	0.7350 (7)	−0.1917 (6)	0.0266 (16)	
C12	0.4125 (9)	1.0184 (7)	0.3422 (6)	0.0262 (15)	
C13	0.5138 (10)	1.1425 (7)	0.3342 (6)	0.0320 (17)	
H13	0.5811	1.1507	0.2722	0.038*	
C14	0.5213 (11)	1.2583 (8)	0.4149 (7)	0.0395 (19)	
H14	0.5901	1.3415	0.4054	0.047*	
C15	0.4263 (11)	1.2479 (8)	0.5081 (6)	0.0366 (19)	
H15	0.4322	1.3244	0.5627	0.044*	
C16	0.3202 (9)	1.1231 (8)	0.5222 (6)	0.0294 (16)	
C17	0.2175 (10)	1.1057 (8)	0.6155 (6)	0.0339 (18)	
H17	0.2185	1.1795	0.6718	0.041*	
C18	0.1181 (10)	0.9824 (8)	0.6233 (6)	0.0350 (18)	
H18	0.0494	0.9702	0.6842	0.042*	
C19	0.1202 (11)	0.8728 (8)	0.5377 (7)	0.0374 (19)	
H19	0.0531	0.7882	0.5451	0.045*	
C20	0.3119 (9)	1.0069 (7)	0.4397 (6)	0.0270 (16)	
C21	0.6964 (12)	0.4626 (8)	0.9114 (7)	0.046 (2)	
H21A	0.7048	0.5297	0.9796	0.069*	
H21B	0.7884	0.4949	0.8650	0.069*	
H21C	0.7047	0.3830	0.9323	0.069*	
C22	0.5275 (15)	0.4318 (9)	0.8452 (8)	0.050 (2)	
N1	0.1179 (7)	0.8547 (5)	0.0109 (4)	0.0225 (12)	
N2	0.2631 (8)	0.8640 (6)	0.1645 (5)	0.0262 (13)	
N3	0.2115 (8)	0.8818 (6)	0.4468 (5)	0.0312 (14)	
N4	0.3994 (14)	0.4115 (9)	0.7958 (7)	0.068 (3)	
Pd1	0.5000	1.0000	0.0000	0.0191 (2)	

### Atomic displacement parameters (Å^2^)

**Table:**

	*U*^11^	*U*^22^	*U*^33^	*U*^12^	*U*^13^	*U*^23^
Br1	0.0317 (5)	0.0264 (4)	0.0355 (5)	0.0164 (3)	0.0098 (3)	0.0090 (3)
C1	0.027 (4)	0.022 (3)	0.020 (3)	0.013 (3)	0.002 (3)	0.003 (3)
C2	0.029 (4)	0.029 (4)	0.029 (4)	0.009 (3)	0.003 (3)	0.007 (3)
C3	0.042 (5)	0.033 (4)	0.026 (4)	0.017 (4)	0.018 (3)	0.013 (3)
C4	0.027 (4)	0.019 (3)	0.024 (4)	0.005 (3)	0.002 (3)	0.005 (3)
C5	0.033 (4)	0.042 (5)	0.021 (4)	0.019 (4)	0.001 (3)	0.011 (3)
C6	0.038 (4)	0.033 (4)	0.024 (4)	0.012 (4)	0.005 (3)	0.008 (3)
C7	0.050 (5)	0.042 (5)	0.039 (5)	0.028 (4)	0.010 (4)	0.009 (4)
C8	0.056 (6)	0.033 (4)	0.033 (4)	0.018 (4)	0.012 (4)	0.003 (4)
C9	0.045 (5)	0.039 (5)	0.026 (4)	0.011 (4)	−0.003 (3)	0.009 (4)
C10	0.038 (4)	0.034 (4)	0.023 (4)	0.016 (4)	0.003 (3)	0.006 (3)
C11	0.031 (4)	0.033 (4)	0.018 (4)	0.012 (3)	0.005 (3)	0.007 (3)
C12	0.026 (4)	0.036 (4)	0.020 (4)	0.014 (3)	−0.005 (3)	0.007 (3)
C13	0.032 (4)	0.040 (5)	0.023 (4)	0.009 (3)	0.005 (3)	0.011 (3)
C14	0.049 (5)	0.035 (5)	0.031 (4)	0.010 (4)	−0.004 (4)	0.005 (4)
C15	0.048 (5)	0.038 (5)	0.026 (4)	0.019 (4)	−0.001 (4)	0.003 (3)
C16	0.031 (4)	0.037 (4)	0.022 (4)	0.013 (3)	0.000 (3)	0.007 (3)
C17	0.045 (5)	0.045 (5)	0.019 (4)	0.025 (4)	0.001 (3)	0.005 (3)
C18	0.046 (5)	0.050 (5)	0.016 (4)	0.025 (4)	0.004 (3)	0.006 (3)
C19	0.051 (5)	0.037 (5)	0.033 (4)	0.021 (4)	0.006 (4)	0.015 (4)
C20	0.029 (4)	0.035 (4)	0.022 (4)	0.014 (3)	0.001 (3)	0.010 (3)
C21	0.059 (6)	0.039 (5)	0.039 (5)	0.016 (4)	0.012 (4)	0.007 (4)
C22	0.084 (8)	0.043 (5)	0.034 (5)	0.035 (6)	0.022 (5)	0.007 (4)
N1	0.025 (3)	0.022 (3)	0.018 (3)	0.006 (2)	−0.002 (2)	0.001 (2)
N2	0.033 (3)	0.029 (3)	0.022 (3)	0.016 (3)	0.006 (3)	0.008 (3)
N3	0.037 (4)	0.038 (4)	0.026 (3)	0.019 (3)	0.003 (3)	0.011 (3)
N4	0.097 (8)	0.060 (6)	0.055 (6)	0.044 (6)	−0.001 (5)	0.004 (4)
Pd1	0.0223 (4)	0.0213 (4)	0.0163 (4)	0.0102 (3)	0.0038 (3)	0.0047 (3)

### Geometric parameters (Å, °)

**Table:**

Br1—Pd1	2.4245 (10)	C10—C11	1.376 (10)
C1—N1	1.344 (8)	C10—H10	0.9300
C1—N2	1.359 (8)	C12—C13	1.362 (10)
C1—Pd1	2.026 (7)	C12—C20	1.437 (9)
C2—C3	1.323 (10)	C13—C14	1.400 (11)
C2—N1	1.384 (8)	C13—H13	0.9300
C2—H2	0.9300	C14—C15	1.369 (11)
C3—N2	1.390 (9)	C14—H14	0.9300
C3—H3	0.9300	C15—C16	1.401 (10)
C4—N1	1.460 (8)	C15—H15	0.9300
C4—C11	1.532 (9)	C16—C17	1.412 (10)
C4—H4A	0.9700	C16—C20	1.412 (10)
C4—H4B	0.9700	C17—C18	1.349 (11)
C5—N2	1.472 (9)	C17—H17	0.9300
C5—C12	1.496 (10)	C18—C19	1.402 (11)
C5—H5A	0.9700	C18—H18	0.9300
C5—H5B	0.9700	C19—N3	1.328 (9)
C6—C7	1.359 (11)	C19—H19	0.9300
C6—C11	1.378 (10)	C20—N3	1.364 (9)
C6—H6	0.9300	C21—C22	1.491 (14)
C7—C8	1.412 (12)	C21—H21A	0.9600
C7—H7	0.9300	C21—H21B	0.9600
C8—C9	1.373 (11)	C21—H21C	0.9600
C8—H8	0.9300	C22—N4	1.132 (13)
C9—C10	1.417 (11)	Pd1—C1^i^	2.026 (7)
C9—H9	0.9300	Pd1—Br1^i^	2.4245 (10)
			
N1—C1—N2	104.4 (6)	C12—C13—H13	118.5
N1—C1—Pd1	128.7 (5)	C14—C13—H13	118.5
N2—C1—Pd1	126.8 (5)	C15—C14—C13	119.4 (8)
C3—C2—N1	106.7 (6)	C15—C14—H14	120.3
C3—C2—H2	126.6	C13—C14—H14	120.3
N1—C2—H2	126.6	C14—C15—C16	120.7 (7)
C2—C3—N2	107.4 (6)	C14—C15—H15	119.7
C2—C3—H3	126.3	C16—C15—H15	119.7
N2—C3—H3	126.3	C15—C16—C17	123.5 (7)
N1—C4—C11	113.1 (5)	C15—C16—C20	119.6 (7)
N1—C4—H4A	109.0	C17—C16—C20	116.9 (7)
C11—C4—H4A	109.0	C18—C17—C16	120.2 (7)
N1—C4—H4B	109.0	C18—C17—H17	119.9
C11—C4—H4B	109.0	C16—C17—H17	119.9
H4A—C4—H4B	107.8	C17—C18—C19	118.7 (7)
N2—C5—C12	111.5 (6)	C17—C18—H18	120.6
N2—C5—H5A	109.3	C19—C18—H18	120.6
C12—C5—H5A	109.3	N3—C19—C18	124.4 (7)
N2—C5—H5B	109.3	N3—C19—H19	117.8
C12—C5—H5B	109.3	C18—C19—H19	117.8
H5A—C5—H5B	108.0	N3—C20—C16	123.3 (6)
C7—C6—C11	122.7 (8)	N3—C20—C12	117.2 (6)
C7—C6—H6	118.7	C16—C20—C12	119.5 (7)
C11—C6—H6	118.7	C22—C21—H21A	109.5
C6—C7—C8	118.6 (8)	C22—C21—H21B	109.5
C6—C7—H7	120.7	H21A—C21—H21B	109.5
C8—C7—H7	120.7	C22—C21—H21C	109.5
C9—C8—C7	120.3 (8)	H21A—C21—H21C	109.5
C9—C8—H8	119.8	H21B—C21—H21C	109.5
C7—C8—H8	119.8	N4—C22—C21	178.4 (10)
C8—C9—C10	119.2 (8)	C1—N1—C2	111.3 (5)
C8—C9—H9	120.4	C1—N1—C4	124.5 (6)
C10—C9—H9	120.4	C2—N1—C4	124.1 (6)
C11—C10—C9	120.2 (7)	C1—N2—C3	110.1 (6)
C11—C10—H10	119.9	C1—N2—C5	124.8 (6)
C9—C10—H10	119.9	C3—N2—C5	125.0 (6)
C10—C11—C6	119.0 (7)	C19—N3—C20	116.5 (7)
C10—C11—C4	119.6 (6)	C1—Pd1—C1^i^	180.000 (1)
C6—C11—C4	121.4 (7)	C1—Pd1—Br1	90.45 (18)
C13—C12—C20	117.9 (7)	C1^i^—Pd1—Br1	89.55 (18)
C13—C12—C5	121.5 (7)	C1—Pd1—Br1^i^	89.55 (18)
C20—C12—C5	120.6 (6)	C1^i^—Pd1—Br1^i^	90.45 (18)
C12—C13—C14	123.0 (7)	Br1—Pd1—Br1^i^	180.0

Symmetry codes: (i) −*x*+1, −*y*+2, −*z*.
